# Promotion of Vape Tricks on YouTube: Content Analysis

**DOI:** 10.2196/12709

**Published:** 2019-06-18

**Authors:** Grace Kong, Heather LaVallee, Alissa Rams, Divya Ramamurthi, Suchitra Krishnan-Sarin

**Affiliations:** 1 Yale School of Medicine New Haven, CT United States; 2 Stanford University Stanford, CA United States

**Keywords:** e-cigarettes, social media, marketing

## Abstract

**Background:**

The ability to perform vape tricks (ie, blowing large vapor clouds or shapes like rings) using e-cigarettes appeals to youth. Vape tricks are promoted on social media, but the promotion of vape tricks on social media is not well understood.

**Objective:**

The aim of this study was to examine how vape tricks were promoted on YouTube to youth.

**Methods:**

Videos on vape tricks that could be accessed by underage youth were identified. The videos were coded for number of views, likes, dislikes, and content (ie, description of vape tricks, e-cigarette devices used for this purpose, video sponsors [private or industry], brand marketing, and contextual characteristics [eg, model characteristics, music, and profanity]).

**Results:**

An analysis of 59 sample videos on vape tricks identified 25 distinct vape tricks. These videos had more likes than dislikes (11 to 1 ratio) and a 32,017 median view count. 48% (28/59) of the videos were posted by industry accounts (27% [16/59] provaping organizations, 15% [9/59] online shops, and 3% [2/59] vape shops) and 53% by private accounts (55% [17/31] private users, 26% [8/31] vape enthusiasts, and 19% [6/31] YouTube influencers); 53% (31/59) of the videos promoted a brand of e-cigarette devices, e-liquids, or online/vape shops, and 99% of the devices used for vape tricks were advanced generation devices. The models in the videos were 80.2% (160/198) male, 51.5% white (102/198), and 61.6% (122/198) aged 18 to 24 years; 85% (50/59) of the videos had electronic dance music and hip hop, and 32% (19/59) had profanity.

**Conclusions:**

Vape trick videos on YouTube, about half of which were industry sponsored, were accessible to youth. Restrictions of e-cigarette marketing on social media, such as YouTube, are needed.

## Introduction

### Background

E-cigarettes have rapidly gained popularity among adolescents and adults in the United States and around the world since their introduction to the market around 2007. US national data show that although current e-cigarette use (defined as using it every day or some days) is 3.3% among adults older than 24 years, use among young adults aged 18 to 24 years is higher at 5.2% [1], and current use (defined as using it one day or more in the past 30 days) is 20.8% among high school students [2].

Understanding the appeal of e-cigarettes is critical to inform e-cigarette prevention efforts aimed at adolescents and young adults. Vape tricks, which involve using e-cigarettes to blow large, thick amounts of exhaled aerosol (ie, clouds) or shapes such as rings, are known to be appealing to youth [3,4]. There are even competitions held with prizes, sponsors, judges, and spectators to see who can produce impressive vape tricks using e-cigarettes [5]. The appeal of vape tricks appears to be unique to youth as this appeal has been identified as a reason for e-cigarette initiation among youth e-cigarette users [3,4] but not among adult users [6]. However, despite the appeal of vape tricks among youth, there is limited information on this e-cigarette use behavior. It is important to understand how vape tricks are conducted and promoted to youth as the appeal of vape tricks could lead youth to initiate e-cigarettes [3,4] and expose them to nicotine dependence and to other unknown health effects from using e-cigarettes.

### Use of Social Media to Understand E-Cigarette Use

Social media provides a unique opportunity to understand health-related behaviors [7] and novel tobacco use behaviors, including e-cigarettes [8]. Social media is particularly an important medium to examine as youth are actively engaging in social media, and importantly, they are exposed to protobacco content through this medium [9]. Youth exposure to protobacco content on social media is concerning owing to its influence on youth behaviors and perceptions.

According to the Social Cognitive Theory of Mass Communication [10], exposure to social media influences youth’s cognition, emotion, and behavior. Thus, positive perceptions regarding e-cigarettes and e-cigarette use behaviors, such as conducting vape tricks, may be perpetuated by e-cigarette-related contents portrayed on social media. Indeed, existing studies have examined e-cigarette-related perceptions and use behaviors on popular social media websites among youth, such as Reddit [11,12], Twitter [13-15], Instagram [16-19], and YouTube [20-22]. Thus, analysis of social media data provides an important means to understand how e-cigarette use behaviors are promoted without being primed by a researcher.

### Use of YouTube to Understand Vape Tricks

In this study, we have focused on YouTube to learn how vape tricks are promoted to youth. YouTube is a free and popular website that allows users to upload and share videos and it is extremely popular among youth. YouTube has over a billion users, and more young people (aged 18 to 34 years) are watching YouTube videos than any cable network channels [23]. Moreover, adolescents who are younger than 18 years are 1.5 times more likely than adults to frequent YouTube [24]. Adolescents are also more likely than adults to be exposed to alcohol and tobacco content on YouTube videos [25].

YouTube is a useful source to learn about traditional and novel tobacco use behaviors. For instance, YouTube has been used to understand unorthodox tobacco use methods, such as techniques on manipulating cigars [26]. YouTube videos are widely used to promote various tobacco products, including cigarettes [27,28], hookah [29], smokeless tobacco [30,31], cigars and little cigars [32], and e-cigarettes [20,22,33-36]. For instance, vape pens have been promoted via music videos on YouTube, and these music videos have been viewed over 1.4 billion times in 24 months [37]. The widespread presence of protobacco content on YouTube is concerning as it can influence perceptions and behaviors of their young viewers. As YouTube is readily available to all age groups, and may have a particularly greater influence on adolescents, it is important to understand if and how this social media platform is being used to promote vape tricks. Youth may turn to YouTube to learn how to conduct new behaviors that they have never conducted before, such as vape tricks [38].

### The Goal of This Study

We examined the content of YouTube videos that promote vape tricks, and which may be accessible to youth, using a nonage verified account. We used search terms related to tutorials and *how to* videos (eg, step-by-step instructions on how to conduct vape tricks) on conducting vape tricks on YouTube to assess the types of information young users may be exposed to. We aimed to describe the availability of these videos, the video characteristics (eg, number of views and *likes*), and the type of vape tricks shown and e-cigarette devices used for this purpose.

Marketing e-cigarettes on social media poses a significant challenge to tobacco control as this content remains relatively unknown. Thus, we determined the source of these videos–whether these videos were posted by the e-cigarette industry (eg, e-cigarette device/e-liquid manufacturers, vape shops, or pro–e-cigarette organizations) or by private users who have no clear links with the e-cigarette industry. We further determined whether industry-sponsored videos had more marketing content than private-user videos. Although it may be evident that industry-sponsored videos would have more marketing content than private-user videos, social media presents novel marketing opportunities for private users to also market products to the public [39-41]. Thus, we also assessed whether private users were YouTube influencers or vape enthusiasts. YouTube influencer is a celebrity who usually posts videos of themselves (eg, vlogging) on a subject matter in which they view themselves as an expert or on other various subject matters through their YouTube channels. They often have a large following and thus possess the potential to reach and market products to many viewers [41,42]. *Vape enthusiasts* are individuals who are experts in vaping and may use social media to promote vaping and market-related products.

We also described contextual video characteristics that could enhance the appeal of these videos to young viewers, such as the production quality of the videos, model characteristics (eg, gender and age), and other appealing aspects such as music. Finally, we assessed the use of profanity in the videos; as these are videos that could also be accessed by youth, the identification of the presence of profanity may be helpful to setting age restrictions to accessing these videos [43].

## Methods

### Procedures

In April/May 2016, we searched for terms related to vape tricks on YouTube, including tutorials/instructions on how to conduct these behaviors (ie, vape tricks, e-cig smoke tricks, how to do vape tricks, how to do smoke tricks with vape, how to do smoke tricks with vape pens, how to do smoke tricks for beginners vape, how to do smoke tricks for beginners e-cigs, vape tricks tutorial, e-cig smoke tricks tutorial, e-cigarette smoke trick tutorial, and vape smoke tricks tutorial). YouTube restricts minors younger than 18 years from videos with inappropriate contents, such as vulgar language, violence and disturbing imagery, nudity/sexually suggestive content, and portrayal of harmful or dangerous activities [44]. To access these videos, viewers must register their date of birth on their Google account. However, users not signed into an age-verified account could access all other videos that are not flagged as inappropriate. Adolescents may sign in from accounts that are not age verified or from age-verified accounts that automatically prohibit them from accessing flagged videos, so we searched for the terms from a YouTube account that was not age verified.

**Figure 1:**
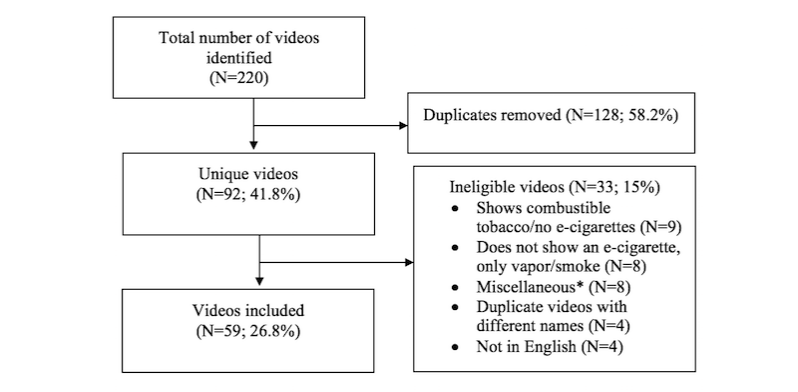
Inclusion of YouTube videos on vape tricks and reasons for exclusion. The videos shown on the first 2 pages of each search term (n=11) were considered for inclusion. *Miscellaneous ineligible videos included videos on magic tricks, how to hack an e-cigarette not for conducting vape tricks, and video games unrelated to vape tricks.

We used search parameters that have been used to examine tobacco use behaviors on YouTube [29,33,45]: (1) the search was limited to videos uploaded in the past year with a duration of <4 min, (2) the videos were sorted by relevance (default sorting option), and (3) videos on the first 2 pages (20 videos) using each of the search terms were downloaded. Previous studies showed that the majority of internet users click on the first page of search results [46]. Based on this finding, studies examining YouTube videos examined the first 20 videos shown on the first 2 pages of the search results with the assumption that users would not watch more than 20 videos [29,33,35]. To be included for analysis, videos had to be in English, show a vape trick, and an e-cigarette to do the vape trick to confirm that the vape tricks were conducted using e-cigarettes and not other tobacco products such as cigarettes or hookah. See Figure 1 for the number of videos identified and reasons for exclusion.

To analyze the videos, a codebook was developed based on a previously conducted analysis of tobacco-related content of YouTube videos [26]. Specifically, we obtained descriptive information about YouTube videos, such as the number of comments, views, likes, dislikes, and duration of the videos. Content analysis included vape trick characteristics (ie, identification and description of vape tricks and e-cigarettes used for this purpose), video source (ie, private vs e-cigarette industry and the presence of marketing content), and contextual characteristics that enhanced the appeal of the videos such as models and music. A detailed description of each content area is provided below.

The final codebook was developed iteratively. These content areas were identified and described in detail by the lead researcher who viewed videos that met the criteria except for the year of publication (ie, videos published 6 months before the specified dates used in search) and then they were confirmed or modified by 2 independent coders who also reviewed the same videos. All coders resolved any differences in coding through discussions. Upon the development of the codebook, we determined interrater reliability by coding 30% of videos not included in the final analysis (ie, videos uploaded 6 months before the specified dates used in search). To establish interrater reliability, 2 independent coders coded 17 videos. The coding was then reviewed by 2 coders and by the leader researcher to amend coding differences and answer any questions that arose during coding. The coders then coded the videos included in this study. Each video was viewed as many times as possible to code all aspects of the interested variables.

### Content Areas

#### Vape Trick Characteristics

*Vape tricks* were identified and described based on the information provided by the models on the YouTube videos and the visual and verbal demonstrations of vape tricks shown on the videos. We described each vape trick and determined how many times each vape trick was shown on all videos. The Cohen kappa was 0.69, *P*<.001.

E-cigarette devices (eg, box mods, vape pens, and cigalikes) used for vape tricks were identified through visual confirmation by the coders. The Cohen kappa was 0.85, *P*<.001.

#### Video Source Characteristics

*Video source* was defined as an e-cigarette industry (eg, e-cigarette or e-liquid manufacturers, online shops, vape shops, or pro–e-cigarette professional organizations) or a private person who did not have a clear affiliation to an e-cigarette industry and appeared to represent only himself/herself. To determine the video source, we examined the channel pages of each YouTube video. The channel pages referred to the profile page of the YouTube user identifier who posted the video. This page could be used to obtain information about the posters, such as their affiliated businesses or organizations, when applicable. Industry-sponsored channels were further categorized into (1) vape shops if the channel was associated with specific brick and mortar stores, (2) Web-based shops if they were selling e-cigarettes or related products online but were not associated with brick and mortar stores, or (3) pro–e-cigarette organizations if they endorsed vaping by providing reviews about e-cigarette devices, vape tricks, or shared other news associated with e-cigarettes but did not sell any tobacco products.

We also assessed whether the videos were posted by a YouTube influencer. YouTube influencer was defined as a single key person who had a significant presence on YouTube with either many subscribers (>10,000) or more than 1 million views of their channels. Another source of influence may be a *vape enthusiast*. We determined *vape enthusiast* on the basis of the description provided on the channel page and if they only included videos on e-cigarettes on their video playlist. The Cohen kappa was 0.92, *P*<.001.

*Marketing* was determined if there was verbal or nonverbal brand promotion of e-cigarette devices, e-liquids, vape shops, or e-cigarette–related organizations. Examples of verbal marketing were a model saying the brand names of the devices or the e-liquids that they were using to conduct vape tricks or acknowledging their sponsors (eg, vape shops) on the video. Examples of nonverbal brand marketing included displaying the brand names or the images of the logo of the devices or the e-liquids or sponsor on the screen, on clothing or hat worn by the models, or on signs and posters in the background. Cohen kappa was 0.63, *P*<.001.

#### Contextual Characteristics

*Video type* was categorized into (1) tutorials: step-by-step instructions on how to conduct vape tricks, (2) compilations: amalgam of video clips of various types of vape tricks in 1 video, and (3) *other*: other types of video that could not be categorized into the specified categories. Cohen kappa was 0.84, *P*<.001.

*Production quality* was determined by ratings of low, moderate, or high based on previously used coding of YouTube videos [29]. The videos were coded low if they looked like they were homemade and there was little or no attention to production values such as lighting, camera angles, and sound quality; moderate if they were homemade but at least some attention was paid to production values; high if the videos were produced with great attention to these production values. Cohen kappa was 0.68, *P*<.001.

*Model characteristics* such as gender, race, and age were determined for each model shown on the video using the coding scheme previously used to code models on smoking videos on YouTube [45]. Gender was coded as male, female, and cannot identify. Perceived race was coded as white (including European), Asian (including Asian-American), black/African American, Latino, and cannot Identify. Perceived age included younger than 18 years, 18 to 24 years, 25 to 34 years, 35 to 59 years, 60 years or older, and cannot identify. Cohen kappa for each demographic variable was 0.92, 0.63, 0.83, *P*<.001, respectively.

*Other contextual elements* such as the use of music and profanity were assessed. We coded the music into categories of electronic dance music (EDM), hip hop, pop, and classical. We also coded for the use of verbal and nonverbal profanity (ie, sticking out the middle finger). Cohen kappa for music was 1.00, *P*<.001, and Cohen kappa for profanity was 0.74, *P*<.001.

## Results

### Video Characteristics

The 11 search terms identified a total of 156,200 videos (mean 14,200 [SD 12,529]; range 2190-43,100). The videos shown only on the first 2 pages (20 videos on each page) for each search term (N=220) were considered. After the removal of duplicate videos (n=128) and the videos that did not meet our inclusion criteria (n=33), 59 videos were included in the final analysis (see Figure 1 for specific reasons for exclusion). Table 1 lists the descriptive statistics of the videos, such as the published date of the videos, duration of the videos, and the number of views, likes, dislikes, and comments. The average length/duration of the videos was 2 min 14 s (SD 55 seconds), and the videos had more *likes* than *dislikes* (11 to 1 ratio).

### Vape Trick Characteristics

#### Vape Tricks

**Figure 2:**
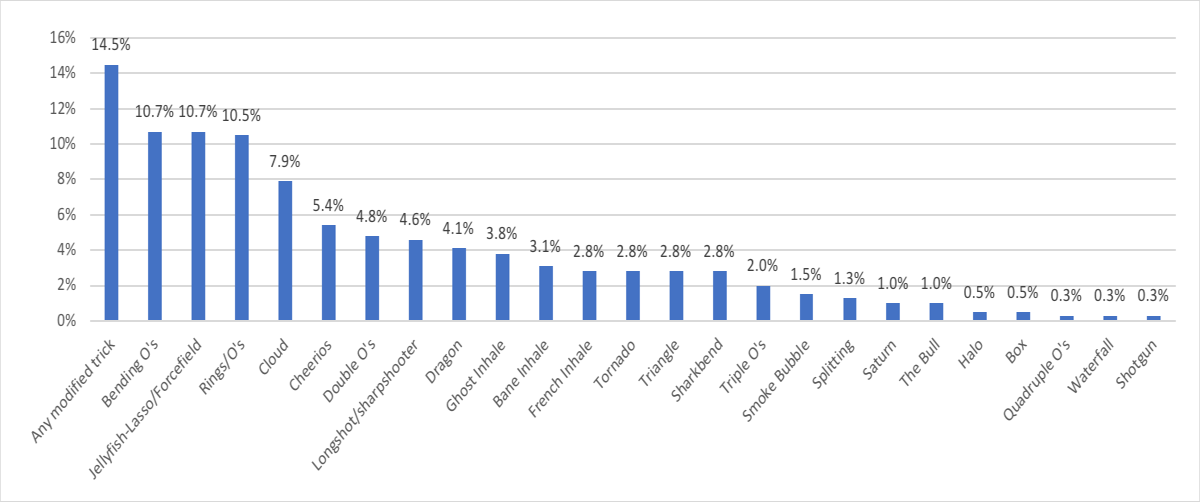
The 25 vape tricks identified in YouTube videos. See Supplemental material for the description of these vape tricks. The frequencies were based on the number of times these vape tricks appeared on all videos. Each video may have featured more than one vape trick.

We identified 25 distinct vape tricks. The median number of vape tricks shown on each video was 5 (interquartile range=4, range 1-20). See Figure 2 for the names of the vape tricks and the frequencies of vape tricks shown on the videos; a further description of these vape tricks was provided in the supplemental materials. The most common vape trick was a *modified trick*, which was a modification of any existing vape trick.

#### E-Cigarette Devices Used for Vape Tricks

**Figure 3:**
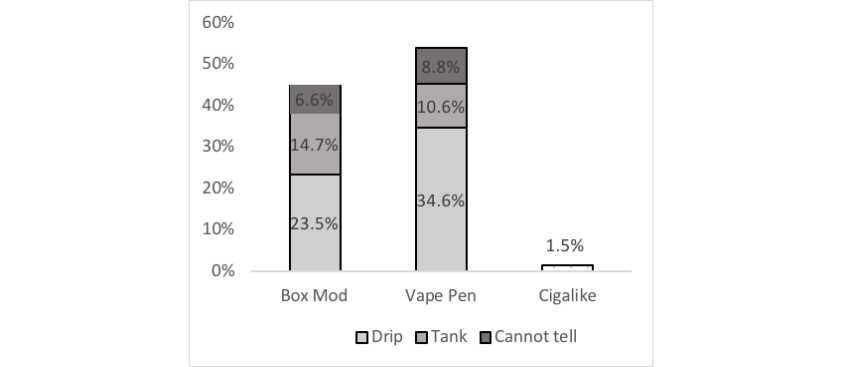
E-cigarette devices used for vape tricks on YouTube videos.

We were able to determine the type of e-cigarette devices for 72.0% (136/189) of the devices shown . A total of 98.5% (134/136) of the devices were advanced generation devices (44.8% box mod (61/136) and 53.7% (73/136) vape pens) and 1.5% (2/136) of the devices were cigalikes. Box mods and vape pens were further categorized into whether they were drip or tank devices (Figure 3).

**Table 1:** Descriptive statistics of vape trick video characteristics.

Video characteristics	Median	Interquartile range	Minimum value	Maximum value
Published date	November 20, 2016	200 days	April 29, 2015	April 13, 2016
Views	32,017	122,842	44	2,331,038
Likes	194	596	1	7439
Dislikes	8	35	0	1787
Duration (Min:Seconds)	2:08	1:30	0:17	3:52
Comments	21	50	0	1384

### Video Source Characteristics

#### Video Source

Personal channels posted 53% (31/59) of the videos and industry-sponsored channels posted 48% (28/59) of the videos (27% (16/59) provaping organizations, 15.3% (9/59) Web-based shops, and 3.4% (2/59) vape shops). Of the private channels, 55% (17/31) were by private user channels, 26% (8/31) by vape enthusiasts, and 19% (6/31) by YouTube influencers. Interestingly, only 1 influencer had videos that showcased only e-cigarette–specific video content whereas the other influencers posted videos on various content areas (eg, tattoo, music videos, humor, and viral videos) including content on e-cigarettes. Of the private channels, 25.8% were by “vape enthusiasts,” and 54.8% were private-user channels.

#### Marketing

53% (31/59) of the videos contained marketing content (25% [15/59] nonverbal only, 5% [3/59] verbal only, and 22% [13/59] both nonverbal and verbal). Some examples of nonverbal brand promotion were models conducting vape tricks while wearing shirts or hats with the brand logo, showing an e-liquid bottle with the brand logo and name, or the brand name was displayed on the screen. An example of verbal promotion included the model verbally encouraging the viewers to purchase the e-liquid or devices.

### Extraneous Video Characteristics

#### Video Type

Video types are as follows: 41% (24/59) of the videos were tutorial, 34% (20/59) were compilations, and 25% (15/59) were other. Other videos included short clips of vape trick competitions, model(s) conducting vape tricks on a stage with special lighting and music, and selfie videos of individuals conducting vape tricks.

#### Production Quality

Production quality are as follows: 25% (15/59) were low, 34% (20/59) were moderate, and 41% (24/59) were high quality. Videos sponsored by the industry were more likely to be of high quality relative to personal videos (personal videos: low quality 42% (13/31), moderate quality 52% (16/31), and high quality 7% (2/31) vs industry videos: low quality 7% (2/28), moderate quality 14% (4/28), and high quality 79% (22/28); *Χ*^2^_2_=31.86; *P*<.001).

#### Model Characteristics

There were 198 models in total: 80.2% (160/198) were male, 18.2% (36/198) were female, and 1.0% (2/198) could not be identified. Race varied: 51.5% (102/198) were coded as white, 17.7% (35/198) as Asian, 17.2% (34/198) as Latino, 3.0% (6/198) as black, and 10.6% (21/198) could not be identified. The age groups were 61.6% (122/198) 18 to 24 years, 26.3% (52/198) 25 to 34 years, 5.1% (10/198) younger than 18 years, 1.0% (2/198) older than 35 years, and 6.1% (12/198) could not be identified.

### Other Contextual Elements

#### Music

A total of 58% (34/59) of the videos had only EDM, 15% (9/59) had EDM and other music, such as hip hop and pop, 14% (8/59) of the videos did not have any music, 12% (7/59) had only hip hop, and 2% (1/59) had classical music.

#### Profanity

A total of 32% (19/59) of the videos contained profanity: 10% (6/59) had verbal profanity, 9% (5/59) had models using nonverbal and visual profanity (ie, sticking out the middle finger), 9% (5/59) had music containing profanity, and 5% (3/59) had multiple sources of profanity (usually a mix of music and verbal profanity).

## Discussion

### Principal Findings

The search of vape tricks and tutorials on vape tricks on YouTube resulted in an average of 14,200 YouTube videos, which were videos that could be accessed by underage youth (<18 years). This finding could serve as an impetus for YouTube to set limits to restrict underage youth from accessing videos on vape tricks. YouTube self-regulates by attempting to limit youth from accessing inappropriate videos that are deemed sexually explicit, harmful or dangerous, violent or graphic, hateful, spams/scams, threats, excessive profanity, and copyrighted [47]. To access these flagged videos, users must use age-verified accounts. Currently, protobacco videos do not fall under the definition of unacceptable materials and are readily available without any regulations. Given that the age of legal tobacco use is 18 years and older (in some places 21 years and older), YouTube may consider videos on e-cigarettes and other tobacco use as mature content and flag these videos so that users with age-verified accounts can access them. Furthermore, one-third of the videos in our study contained profanity, which further suggests that these videos are not suitable for young viewers.

The evaluation of sample videos on vape tricks identified 25 distinct vape tricks, which were mostly performed by young adults using advanced generation e-cigarette devices. Advanced generation devices are rechargeable and are highly customizable; they include vape pens, box mods, and mech-mods. In addition, some of these devices have specific features for dripping (ie, applying drops of e-liquid solution onto an atomizer to saturate the wick before coil heating) [48]. The promotion of advanced generation devices, including dripping to conduct vape tricks on YouTube, is concerning as studies have shown that adolescent and young adult e-cigarette users primarily use advanced generation e-cigarettes [49,50], whereas adult users use cigalikes [51]. Moreover, data from Connecticut showed that 26% of adolescent e-cigarette users are dripping [50]. Advanced generation e-cigarette devices may be popular among youth because of the ability to customize the product to drip and also to change the temperature of the devices and the constituents of the e-liquid, such as the flavors, nicotine level, and the ratio of the solvent (propylene glycol [PG] and vegetable glycerin [VG]). These components could be modified to conduct vape tricks. For example, temperatures of the devices and PG/VG ratio of the e-liquids could be modified to produce a large amount of exhaled aerosol to conduct vape tricks.

Conducting vape tricks using advanced generation devices is problematic as the use of these devices can be harmful to users because the increased level of temperature and the higher PG levels are associated with a greater exposure to nicotine and toxicants [52,53]. Furthermore, individuals engaging in vape tricks may alter how they puff by taking in deeper and more frequent puffs, which may also increase health risks. Another concern is the exposure to nicotine dependence and other tobacco use behaviors by conducting vape tricks. For example, youth may be drawn to e-cigarettes via YouTube videos displaying appealing vape tricks and they may practice and use e-cigarettes more frequently to learn how to conduct certain vape tricks, which may expose them to more nicotine and other chemicals in the e-cigarette. However, the vape trick videos on YouTube evaluated in this study did not have information on how to manipulate the devices or to change puffing behaviors to conduct various vape tricks. Future studies should assess which and how e-cigarettes are being customized to conduct vape tricks and if puffing behaviors are altered to assess potential health risks for engaging in this behavior.

We also observed that about half of the YouTube videos that promoted vape tricks were sponsored by the e-cigarette industry, with pro–e-cigarette organizations (which are organizations that promote and advocate for pro–e-cigarette–related issues and policies [54]) being the most common sponsor, followed by online shops and vape shops. These findings suggest that when trying to understand the e-cigarette industry, it is important to recognize that stakeholders such as pro–e-cigarette organizations, online retailers, and vape shops are actively engaging in marketing and promoting e-cigarettes. It is important to note that online shops were coded if they did not have a brick and mortar store associated with the shop. However, it is likely that other organizations and brick and mortar shops had online purchasing options.

The large presence of sponsored videos is consistent with the emerging literature showing that Web 2.0 is the optimal platform for the industry to market their tobacco products because of the ability to reach large number of people at fast speed and at low cost [40]. Promoting vape tricks may be a marketing strategy used by the e-cigarette industry. Vape trick videos are used to promote brands of e-liquids, devices, and vape shops, both verbally and nonverbally. Nonverbal promotion is an inconspicuous way to market as the brands are shown on the models’ clothing, background, devices, or on the screen while the model is conducting vape tricks, and this subtle strategy is an effective marketing strategy [55]. Although verbal and nonverbal promotion is one clear way in which e-cigarettes are marketed, future studies should examine a greater number of videos to understand nuanced marketing strategies used in both industry-sponsored and private videos, using both verbal and nonverbal strategies.

One novel marketing strategy used on social media is the use of YouTube influencers or vaping enthusiasts to promote e-cigarette use and market-associated tobacco products. Indeed, we found that vape trick videos were uploaded by these private users. We also observed that 39% of the videos posted by private users (which includes YouTube influencers and vape enthusiasts) also promoted a specific brand of e-liquid or e-cigarette devices. As e-cigarette marketing on social media continues to grow and evolve, more research is needed on the marketing strategies used on social media platforms to inform restrictions on tobacco marketing.

In addition to the type of vape tricks being conducted, and the devices being used for this purpose, the videos had contextual factors that could be appealing to youth. Identification of such factors may reveal how the appeal of vape tricks is enhanced. Despite using various terms related to tutorials on vape tricks, we found that 59% of the videos did not contain information on how to conduct vape tricks but showcased vape tricks in various ways, such as a compilation of videos clips of vape tricks into 1 video accompanied by music, similar to a music video; individual(s) performing vape tricks on a stage accompanied by special lighting and music; vape trick competition videos, which were mostly brief summaries of vape trick competitions featuring winners and their featured vape trick and *news reporters* interviewing the winners; and *selfie* videos where a user self-records himself/herself conducting vape tricks. These various methods of showcasing vape tricks could enhance the appeal of e-cigarettes to youth.

Interestingly, our examination of YouTube videos shows that vape tricks are being conducted as competitions. Future studies are needed to assess the prevalence of these competitions, who attends them, who promotes them, and how these competitions are used to promote e-cigarettes.

The identified contextual factors of YouTube videos may contribute to the coolness of conducting vape tricks. Research on perceptions has shown that short-term benefits such as looking cool are associated with tobacco use and intent to use tobacco [56]. Even though the self-report data suggest that vape tricks are appealing to adolescents [3,4], data on youth’s perceptions of vape trick videos on YouTube are lacking. However, the high number of views and likes does suggest popularity. In fact, the view count for vape trick videos identified in this study was comparable with previous research on videos on e-cigarettes [22] and smokeless tobacco [30] identified on YouTube.

It is interesting to note that 80% of the models in the videos were males. Although national data have shown that e-cigarette use is greater among male adolescents and adults [57,58], whether vape tricks are more popular among males is unknown. Although it is possible that e-cigarette use and associated behaviors such as vape tricks are more common among males, it is also possible that males are more likely to post videos of themselves conducting vape tricks than females. Sex difference in e-cigarette use and conducting vape tricks using e-cigarettes need further investigation.

### Limitations

There are several study limitations. First, we used terms related to vape trick tutorials to assess basic videos that novel users may find on YouTube. There are other terms used to refer to vape tricks, such as cloud chasing or plume tricks that were not used in this study, so the vape tricks identified in this study are not an exhaustive list. Second, we only examined YouTube and did not examine other social media platforms. Other social media platforms such as Facebook, Instagram, Twitter, and Reddit can be used to promote vape tricks and need to be examined, as they have been used to relay information about e-cigarettes [16,59]. Third, we cannot determine whether conducting vape tricks increases the risk for developing nicotine dependence and engaging in other problematic tobacco use outcomes. Future studies need to examine the role of vape tricks on initiation and progression of e-cigarette use behaviors among youth. Youth may be experimenting with vape tricks and eventually stop once the novelty wears off or this behavior may lead to continual future use of the product and expose users to harm and other tobacco use. Fourth, we cannot determine whether youth are exposed to greater health risks by engaging in vape tricks through our study. Although our study findings did show that 99% of the vape tricks were conducted using advanced generation devices, future studies also need to examine whether youth engaging in vape tricks are engaging in riskier e-cigarette use behaviors (eg, dripping). Fifth, the kappa scores ranged from moderate to almost perfect [60]. Although these kappa values are acceptable, we did not conduct another interrater reliability after the discussion to amend any discrepancies. Future studies should conduct interrater reliability before and after the discussion to ensure that the coders are coding consistently. Finally, we did not assess how the contextual features in these vape trick videos appeal to real youth; future studies should examine which aspect of conducting vape tricks and video contextual features that are identified appeals to youth to directly assess the impact of these videos on enhancing the appeal of e-cigarettes.

### Conclusions

This study also suggests areas for future e-cigarette prevention and education efforts. If YouTube videos are used to teach users about vape tricks and other e-cigarette–related information, then YouTube may be also used to disseminate health and risk information about e-cigarettes.

## Abbreviations

EDM: electronic dance music
FDA: Food and Drug Administration
NIDA: National Institute on Drug Abuse
NIH: National Institutes of Health
PG: propylene glycol
VG: vegetable glycerin
